# The Effect of Blood Lipids on the Left Ventricle: A Mendelian Randomization Study

**DOI:** 10.1016/j.jacc.2020.09.583

**Published:** 2020-11-24

**Authors:** Nay Aung, Mihir M. Sanghvi, Stefan K. Piechnik, Stefan Neubauer, Patricia B. Munroe, Steffen E. Petersen

**Affiliations:** a William Harvey Research Institute, Queen Mary University of London, London, United Kingdom; b National Institute for Health Research Barts Cardiovascular Biomedical Research Centre, Queen Mary University of London, London, United Kingdom; c Barts Heart Centre, St. Bartholomew’s Hospital, Barts Health NHS Trust, London, United Kingdom; d Division of Cardiovascular Medicine, Radcliffe Department of Medicine, University of Oxford, Oxford, United Kingdom

**Keywords:** cardiac remodeling, cardiovascular risk, cholesterol, lipids, Mendelian randomization

## Abstract

**Background:**

Cholesterol and triglycerides are among the most well-known risk factors for cardiovascular disease.

**Objectives:**

This study investigated whether higher low-density lipoprotein (LDL) cholesterol and triglyceride levels and lower high-density lipoprotein cholesterol level are causal risk factors for changes in prognostically important left ventricular (LV) parameters.

**Methods:**

One-sample Mendelian randomization (MR) of 17,311 European individuals from the UK Biobank with paired lipid and cardiovascular magnetic resonance data was performed. Two-sample MR was performed by using summary-level data from the Global Lipid Genetics Consortium (n = 188,577) and UK Biobank Cardiovascular Magnetic Resonance substudy (n = 16,923) for sensitivity analyses.

**Results:**

In 1-sample MR analysis, higher LDL cholesterol was causally associated with higher LV end-diastolic volume (β = 1.85 ml; 95% confidence interval [CI]: 0.59 to 3.14 ml; p = 0.004) and higher LV mass (β = 0.81 g; 95% CI: 0.11 to 1.51 g; p = 0.023) and triglycerides with higher LV mass (β = 1.37 g; 95% CI: 0.45 to 2.3 g; p = 0.004). High-density lipoprotein cholesterol had no significant association with any LV parameter. Similar results were obtained by using 2-sample MR. Observational analyses were frequently discordant with those derived from MR.

**Conclusions:**

MR analysis demonstrates that LDL cholesterol and triglycerides are associated with adverse changes in cardiac structure and function, in particular in relation to LV mass. These findings suggest that LDL cholesterol and triglycerides may have a causal effect in influencing cardiac morphology in addition to their established role in atherosclerosis. (J Am Coll Cardiol 2020;76:2477-88).

Both the incidence and prevalence of ischemic heart disease, as well as its long-term sequelae such as heart failure, are on the rise (1,2). Cardiac imaging is an important and widely used tool in guiding the diagnosis and treatment of these patients (3). Left ventricular (LV) parameters derived from cardiac imaging modalities such as end-diastolic volume, ejection fraction, and mass are known to be prognostically important with respect to subsequent major adverse cardiovascular events and cardiovascular death (4,5). Low-density lipoprotein (LDL) cholesterol is one of the best publicized and most unequivocally implicated risk factors in the development of ischemic heart disease; its causal involvement in atherosclerotic plaque formation in the arterial system is well elucidated (6). For triglycerides, a causal relationship with cardiovascular disease has also been demonstrated (7), and for high-density lipoprotein (HDL) cholesterol, low levels are associated with increased risk of cardiovascular disease, but causality has not been established (8). However, to our knowledge, no study has established the causative impact of lipids on the structure and function of the LV.

Mendelian randomization (MR) is an analysis methodology whereby genetic variants associated with a proposed risk factor (e.g., raised LDL cholesterol) are used as surrogates to make causal inferences about the effect of that exposure on an outcome of interest (i.e., LV phenotypes). Given that none of the landmark randomized controlled trials assessing the effect of statins on lipid-lowering and cardiovascular outcomes included cardiac imaging in their protocols, examining the association between cholesterol and LV parameters would traditionally be performed via an epidemiological observational study. Through adopting an MR approach, however, typical biases encountered in observational settings, such as confounding and reverse causation, are mitigated against. With the availability of genotype and cardiovascular magnetic resonance (CMR) data from the UK Biobank as well as large-scale genome-wide association studies for lipids (9) and LV phenotypes (10), examining the causal relationship between lipid concentrations and prognostically important and routinely measured imaging phenotypes has been made possible.

This study investigates whether higher LDL cholesterol and triglycerides and lower HDL cholesterol are causal for changes in LV parameters via individual-level instrumental variable analysis with subsequent sensitivity analysis using summary-level genome-wide association data, to gain further understanding of lipids as cardiovascular risk factors.

## Methods

### Study Cohorts

The UK Biobank is a large population-based, prospective cohort study of 500,000 individuals aged between 40 to 69 years at the time of initial recruitment between 2006 and 2010. It has collected information on health and lifestyle data, physical measurements, biological samples, genotype, and cardiac phenotypes derived from CMR.

The overall study protocol has been described in detail previously (11), as has the CMR protocol and reference ranges (12,13). Genotypes called by the bespoke, closely related UK BiLEVE Axiom and UK Biobank Axiom microarrays (Affymetrix, Santa Clara, California) were imputed by using the Haplotype Reference Consortium and merged UK10K and 1000 Genomes phase 3 reference panels.

Biological samples for biochemical and genetic analysis were taken from participants at their initial baseline visit between 2006 and 2010. CMR examinations, as part of the UK Biobank imaging enhancement, have been performed from 2015 onward.

This study was covered by the general ethical approval for UK Biobank studies from the National Health Service National Research Ethics Service (June 17, 2011 [reference 11/NW/0382]; extended on May 10, 2016 [reference 16/NW/0274]).

### Lipid Measurements

Direct LDL cholesterol serum concentration was measured by enzymatic protective selection analysis on a Beckman Coulter (Brea, California) AU5800 clinical chemistry analyzer. For participants for whom direct measurements were missing, LDL cholesterol concentration was derived by using the Friedewald calculation as long as serum triglyceride concentration was ≤155 mg/dl (4 mmol/l) (14). Where participants had indicated that they used lipid-lowering medications (UK Biobank field ID 20003), LDL cholesterol values were multiplied by a factor of 1.43 to estimate untreated LDL cholesterol serum concentration (15).

Serum HDL cholesterol concentration was measured by the enzyme immune-inhibition method, and serum triglyceride concentration was measured by using a series of coupled enzymatic reactions, both on a Beckman Coulter AU5800 clinical chemistry analyzer.

### Variant Selection And Genetic Risk Score Construction

A weighted genetic risk score (GRS) for LDL cholesterol was built by using variants associated with LDL cholesterol attaining genome-wide significance (p <5 × 10^-8^) reported in the data from the Global Lipids Genetic Consortium (GLGC) (9). Following linkage disequilibrium clumping (at r^2^ < 0.01), 101 independent variants were included in the GRS. Equivalent processes were performed in the same dataset for HDL cholesterol and triglycerides, yielding 125 and 73 variants, respectively (Supplemental Tables 1 to 3). The weighted GRS was calculated for each UK Biobank participant of European ancestry by summing the product of the effect sizes and the number of effect alleles across all selected variants. Variance explained by the weighted GRS was calculated by regressing the measured lipid values on their corresponding GRS. Correlation between the lipid genetic risk scores was assessed by the Pearson test.

### statistical analysis

#### Baseline data and observational analysis

Baseline data are presented in categorized fashion, with participants grouped into unequal bins based on their serum LDL cholesterol percentile. To examine trends across the groups, Cuzick’s extension of the Wilcoxon rank sum test was used for continuous variables, and the chisquare test for trend was used for ordinal variables. CMR parameters used as dependent variables were LV end-diastolic volume (EDV), LV mass, and LV ejection fraction (EF). Non-European ancestries were excluded to improve the homogeneity of the study population and align with the genetic analyses.

To observationally examine the association between phenotypic lipid concentration on important LV parameters, multivariable linear regression models were fitted for each dependent variable. Covariates included age at recruitment, sex, log-transformed body mass index (BMI), body surface area (BSA) (calculated via Dubois and Dubois equation), systolic blood pressure adjusted for antihypertensive medication use (by adding 15 mm Hg) (16), physical activity as determined by log-transformed total metabolic equivalent of task (MET) minutes per week, smoking status, log-transformed glycated hemoglobin (Hb) A1c, and presence of cardiovascular disease (defined as participants diagnosed or reporting myocardial infarction, angina, heart failure, arrhythmias [including atrial fibrillation], cardiomyopathy, stroke, or peripheral vascular disease).

### Instrumental variable analysis

MR was performed by using the 2-stage least squares method (1-sample MR) as implemented in the R package ivpack (R Foundation for Statistical Computing, Vienna, Austria). We included age, sex, BSA, and the first 5 genetic principal components as covariates. Data are presented as the change in LV phenotype per 39-mg/dl (1-mmol/l) increment in lifetime LDL cholesterol and HDL cholesterol exposure and per 89 mg/dl (1 mmol/l) in lifetime triglyceride exposure. Significant causal associations between each lipid GRS and the LV phenotype were additionally tested for the presence of an independent effect by including all 3 lipid genetic risk scores in the regression model. We assessed the presence of weak instrument bias (also known as violation of relevance assumption in MR) by calculating the F-statistic from the linear regression between GRS and LV phenotype. The Durbin-Wu-Hausman test of regressor endogeneity was performed to assess the consistency of the estimate of LV parameter change provided by the instrumental variable analysis compared to the observational analysis. The statistical power of instrumental variable analysis was estimated according to the method proposed by Brion et al. (17). At our available sample size of approximately 17,000 individuals, our 1-sample MR analyses were powered at 80% (alpha = 0.05) to detect the minimum effect sizes of 0.39 to 0.53 ml for LVEDV, 0.16% to 0.24% for LVEF, and 0.31 to 0.45 g for LV mass (Supplemental Figure 1).

### Sensitivity Analyses

As sensitivity analysis to examine the potential causal relationship between serum lipids and prognostically important LV parameters, a 2-sample MR with summary-level genome-wide association data from the GLGC (n = 188,577) (9) and UK Biobank CMR substudy (n = 16,923) (10) was performed. An MR effect estimate for each LV parameter was calculated by the inverse variance-weighted method with robust penalized regression to minimize the influence of genetic variants with outlying ratio estimates (18). Two-sample MR effect sizes are presented as the change in LV parameter per 1-SD increase in LDL cholesterol (34 mg/dl [0.87 mmol/l]), HDL cholesterol (15 mg/dl [0.38 mmol/l]) and triglycerides (90 mg/dl [1.02 mmol/l]), respectively.

Additionally, we used the robust penalized MR-Egger, weighted median, and weighted mode methods to evaluate the validity of genetic instruments (18,19). We assessed the presence of directional horizontal pleiotropy by conducting the MR-Egger intercept test for which a p value of <0.10 was considered as evidence of pleiotropic bias (20). We also conducted the MR pleiotropy residual sum and outlier for further evaluation of horizontal pleiotropy. This test applies 3 procedures: 1) detection of horizontal pleiotropy with the global test; 2) correction for horizontal pleiotropy by outlier removal, known as the outlier test; and 3) assessing significant differences in the causal estimates before and after correction for outliers by using the distortion test. Additionally, we conducted multivariable MR to establish the potential causal effect on LV parameters independent of the effects of the other lipid fractions (21).

As further sensitivity analysis, we performed 1-sample MR following additional adjustment for all covariates (age at recruitment, sex, BMI, BSA, systolic blood pressure adjusted for antihypertensive medication use, physical activity, smoking status, HbA1c, and presence of cardiovascular disease). We built models examining the association between lipid parameters and LV phenotypes using both phenotypic and genetically determined lipid levels as covariates to examine any attenuation effect. We also interrogated the GWAS (Genome-Wide Association Study Catalog) database to identify the variants included in the lipid GRSs that were associated with other lipid and nonlipid traits at a genome-wide significance level. We manually examined this list and excluded the variants associated with traits (e.g., cardiovascular disease, cardiovascular risk factors) that might influence LV remodeling (Supplemental Appendix) and performed the analysis using a restricted GRS.

We investigated the direction of causality by the MR-Steiger test, which is based on the absolute correlations of the genetic variants with the exposure and outcome. The 2-sample MR analyses were conducted using the MendelianRandomization and TwoSampleMR R packages.

### Statin Effect

To examine whether statin use modified the relationship of phenotypic (measured) LDL cholesterol and the LDL GRS, interaction analysis was performed using “statin use × standardized genetic risk score” as an interaction term. For this analysis, we used the LDL cholesterol measurements unadjusted for statin use. We also investigated the effect modification by statin therapy on the association between the LDL GRS and LV parameters.

The causal effects were considered significant only if supported by both 1-sample and 2-sample MR analyses at p < 0.05. All analyses were conducted in the R (3.6.0) statistical computing environment.

## Results

Demographic and clinical characteristics of study participants, as well as median CMR parameter values, are outlined in Table 1. There were 436,064 individuals for whom cholesterol data were available; of these, 17,311 had CMR examinations. Individuals in the top decile for phenotypic LDL cholesterol were older and predominantly female, and they had higher BMI, blood pressure, and HbA1c measurements. The variances in lipid measurements explained by the corresponding genetic risk scores were 10.8% (F-statistic: 2,492), 7.3% (F-statistic: 1,811), and 5.0% (F-statistic: 925) for LDL cholesterol, HDL cholesterol, and triglycerides, respectively. Large F-statistic values (>10) indicated that the MR analyses were unlikely to be affected by the weak instrument bias. The strength of correlations between lipid genetic risk scores was low (Pearson r = 0.10 to -0.26; p > 0.10) (Supplemental Figure 2).

### Ldl Cholesterol

In observational analysis (Table 2, Central Illustration), a 39-mg/dl (1-mmol/l) increase in LDL cholesterol levels was associated with lower LVEDV (β = -2.44 ml; 95% confidence interval [CI]: -2.91 to -1.97 ml; p < 0.0001), lower LV mass (β = -0.64 g; 95% CI: -0.90 to -0.38 g; p < 0.0001), and higher LVEF (β = 0.13%; 95% CI: 0.01 to 0.24%; p = 0.03). In contrast, in 1-sample MR analysis, a 39-mg/dl (1-mmol/l) increase in lifetime LDL cholesterol exposure was associated with higher LVEDV (β = 1.85 ml; 95% CI: 0.59 to3.14 ml; p = 0.004) and higher LV mass (β = 0.81 g; 95% CI: 0.11 to 1.51 g; p = 0.023); there was no significant change in EF. Analyses controlling for HDL and triglycerides genetic instruments did not change the significant results (Supplemental Table 4), indicating that the causal relationships between LDL cholesterol and LVEDV and LV mass were robust to confounding from other lipid fractions. One-sample MR was additionally performed following adjustment for all covariates and yielded similar results (which are detailed, along with results for HDL and triglycerides, in Supplemental Table 5). Models examining the association between LV parameters and genetically determined lipid levels where phenotypic lipid levels were included as a covariate showed no significant attenuation of these associations, indicating an independent effect (Supplemental Table 6). Sensitivity analysis performed using a restricted list of variants in the GRS following exclusion of potentially pleiotropic variants (69, 81, and 50 variants remained for LDL, HDL, and triglycerides, respectively) yielded concordant results to the primary analysis (Supplemental Table 7).

In sensitivity analysis using 2-sample MR (Table 3, Central Illustration), concordant associations were noted for LVEDV (inverse-variance-weighted [IVW] β = 1.62 ml; 95% CI: 0.32 to 2.91 ml; p = 0.014) and LV mass (IVW β = 0.66 g; 95% CI: 0.10 to 1.22 g; p = 0.021). Again, there was no association demonstrated for LVEF. These results were also confirmed by using multivariable MR (Supplemental Table 8). Based on Egger intercept p-values, no directional horizontal pleiotropy was detected. Sensitivity analyses by MR-Egger, weighted median, and weighted mode methods produced associations with concordant effect directions, although the confidence intervals were much wider, as expected (Supplemental Table 9, Supplemental Figure 3). The MR pleiotropy residual sum and outlier method found evidence of horizontal pleiotropy for the association between LDL cholesterol and LVEDV, but upon removal of outlier variants, the effect estimates were not significantly changed as per the distortion tests (Supplemental Table 10). The MR-Steiger test suggested that the assumption of causal directionality for the relationships between the LDL GRS and the LV parameters was correct (Supplemental Table 11). The variance of phenotypic LV parameters explained by observed lipid measurements and lipid genetic risk scores is presented in Supplemental Table 12.

### Hdl Cholesterol

In multivariate analysis, higher phenotypic HDL cholesterol levels were associated with higher LVEDV (β = 8.27 ml; 95% CI: 7.00 to 9.53 ml; p < 0.0001) and higher LV mass (β = 1.34 g; 95% CI: 0.64 to 2.04 g; p = 0.0002), with no association with LVEF. Associations demonstrated in observational analysis were not borne out in 1-sample MR analysis, with no association demonstrated between genetically higher lifetime exposure to HDL cholesterol and changes in LVEDV, LVEF, or LV mass. This was further demonstrated by using summary data in 2-sample MR (Table 3, Central Illustration). These results were reproduced in sensitivity analyses with MR-Egger, weighted median and weighted mode methods (Supplemental Table 9, Supplemental Figure 4).

### Triglycerides

Examining the effect of triglycerides on CMR parameters, observational analysis indicated that an 89-mg/dl (1-mmol/l) increase in triglyceride concentration was associated with lower LVEDV (β = -3.98 ml; 95% CI: -4.40 to -3.55 ml; p < 0.0001), higher LVEF (β = 0.12%; 95% CI: 0.02 to 0.22%; p = 0.024), and lower LV mass (β = -0.65 g; 95% CI: -0.89 to -0.42 g; p < 0.0001). One-sample MR analysis demonstrated that there was no association with changes in LVEDV, but an 89-mg/dl (1-mmol/l) increase in lifetime triglyceride exposure yielded a reduction in LVEF (β = -0.52%; 95% CI: -0.92 to -0.13%; p = 0.011) and higher LV mass (β = 1.37 g; 95% CI: 0.45 to 2.3 g; p = 0.004). Additional adjustment for HDL and triglycerides genetic instruments produced similar results (Supplemental Table 4). Sensitivity analysis performed by using a restricted list of variants in the GRS following the exclusion of potentially pleiotropic variants yielded concordant results to the primary analysis (Supplemental Table 7).

Two-sample MR sensitivity analysis demonstrated no significant association between triglyceride concentration and LVEDV or LVEF; it showed concordant results for LV mass (IVW β = 0.61 g; 95% CI: 0.04 to 1.18 g; p = 0.036). Similar results were observed in multivariable MR analysis (Supplemental Table 8). There was evidence of horizontal pleiotropy for the association between triglycerides and LVEDV, but removal of the outlier variants did not significantly change the effect estimates as indicated by the distortion tests (Supplemental Table 10). Sensitivity analysis examining the association of triglycerides and LV mass using MR-Egger, weighted median, and weighted mode methods did not reach significance because of wider CIs, although they showed concordant effect directions (Supplemental Table 9, Supplemental Figure 5). The assessment of causal directionality using the MR Steiger test supported what this study’s hypothesis has proposed (Supplemental Table 11).

### Statin Use

**Figure 1:**
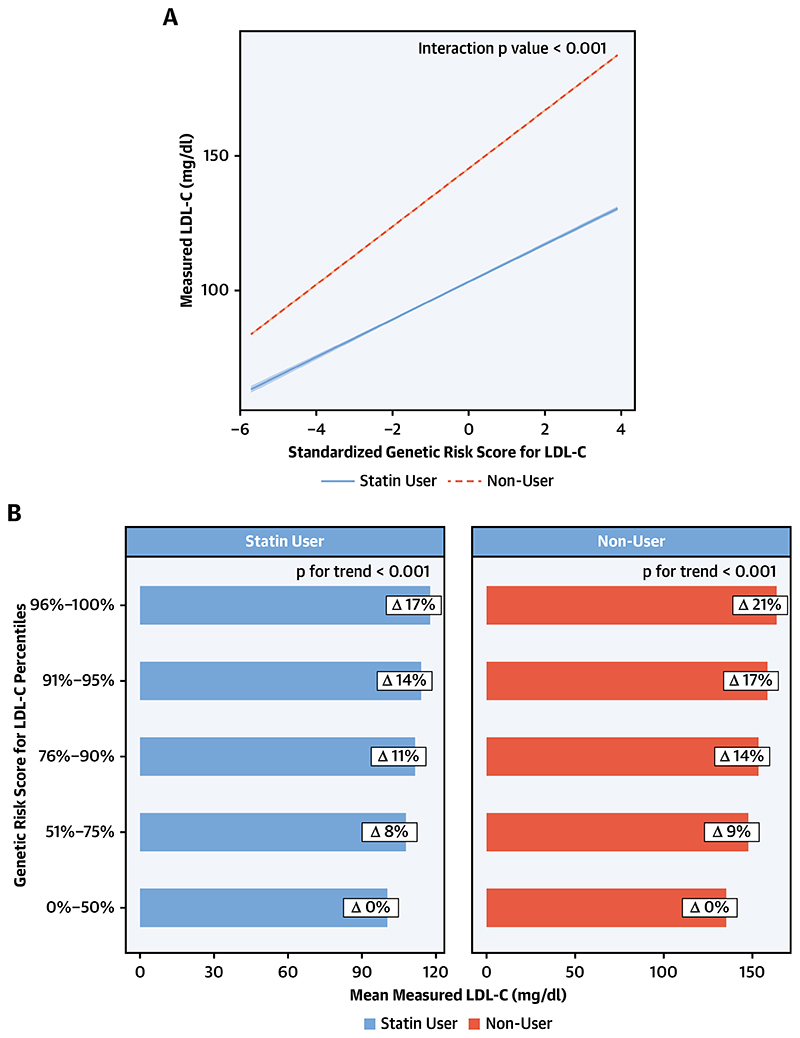
Relationship Between Genetic Risk Score for LDL Cholesterol and Phenotypic LDL Cholesterol by Statin Use (A) Statin use significantly modifies (p for interaction <0.0001) the relationship between measured (phenotypic) LDL cholesterol and the standardized genetic risk score for LDL cholesterol, with statin users exhibiting a reduced measured LDL cholesterol for a given degree of genetic risk. (B) As genetic risk score percentile group increases, the relative increase in measured LDL cholesterol is greater in each group in non-statin users compared to statin users. EDV = end-diastolic volume; EF = ejection fraction; HDL = high-density lipoprotein; LDL = low-density lipoprotein; LV = left ventricle; MR = Mendelian randomization.

To ascertain the effect of statin use, the relationship between measured (phenotypic) LDL cholesterol and the standardized genetic risk score for LDL cholesterol was examined based on whether an individual was a statin user or not. Statin use significantly modified (p for interaction <0.0001) the relationship between measured (phenotypic) LDL cholesterol and the standardized genetic risk score for LDL cholesterol, with statin users exhibiting a reduced measured LDL cholesterol for a given degree of genetic risk (Figure 1A). As demonstrated in Figure 1B, as genetic risk score percentile group increases, the relative increase in measured LDL cholesterol is greater in each group in non-statin users compared to statin users. Examination of the effect modification of statin therapy on the relationships between genetically determined LDL and LV parameters did not yield any significant results (Supplemental Table 13).

## Discussion

To our knowledge, this study is the first to conduct MR analyses to examine the effect of lipids in the development of changes in prognostically important LV parameters. Using instrumental variable analysis in 17,311 individuals with paired genotype and CMR data, with subsequent sensitivity analysis using summary-level data, we demonstrate an association between increased LDL cholesterol and higher LVEDV as well as an association of LV mass and triglycerides with higher LV mass, whereas HDL cholesterol does not result in any significant alterations in LV structure and function. Importantly, results derived from observational analysis were frequently discordant from those obtained via MR.

### Lipids As A Risk Factor: Beyond Atherosclerosis

The substantial body of evidence indicating a continuous, positive, and graded relationship between LDL cholesterol and cardiovascular mortality make cholesterol measurement and the prescription of lipid-lowering therapy cornerstones of primary and secondary prevention in cardiovascular disease (22). LV remodeling is a clinical characterization of the development and progression of morphological changes in the LV that result in ventricular dysfunction (23). These morphological changes have been shown to occur in association with exposure to other important risk factors, such as hypertension (24) or raised body mass index (25), and they are frequently subclinical−present before any discrete clinical event.

In this study, MR analysis demonstrates that both LDL cholesterol and triglycerides have a potentially causal association with increased LV mass. The importance of LV mass as a biomarker in cardiovascular disease is demonstrated in studies where therapeutic interventions that result in a reduction in LV mass have decreased the number of cardiovascular events (26). Importantly, raised LV mass has also been shown to increase the risk of incident heart failure, even in patients free of known ischemic heart disease or previous myocardial infarction—conditions that are atherosclerosis driven (27). LDL cholesterol and triglycerides appear to be causative of myocardial remodeling by increasing LV mass, suggesting that they influence the development of cardiovascular disease not only by atherosclerosis but also by causing adverse alterations in cardiac structure and function.

An insight into the potential mechanistic pathways by which lipids might generate these alterations can be gleaned from work examining the pleiotropic effects of statins on the mevalonate pathway. The mevalonate pathway is a ubiquitous, negative feedback-controlled pathway responsible for cholesterol synthesis; statins act to inhibit cholesterol synthesis by preventing the conversion of HMG-CoA to mevalonate. However, because mevalonate is not the immediate precursor of cholesterol and also acts as a precursor for several other molecules, its inhibition leads to pleiotropic effects being observed, particularly through inhibition of synthesis of isoprenoid intermediates of the mevalonate pathway such as farnesylpyrophosphate and ger-anylgernanylphosphate. An important function of these isoprenoids is the post-translational modification of many guanosine triphosphate-binding proteins of the Rho family (28) of signaling proteins. Rho proteins have been shown to mediate the development of cardiac hypertrophy via a number of mechanisms (29). For example, RhoA is involved in the formation of actin stress fibers and focal adhesion complexes through Rho kinase activation and myosin light chain phosphorylation (30). Rac1 and Cdc42 regulate actin cytoskeletal processes called lamellipodia and filopodia, which are thought to contribute to morphological changes associated with LV hypertrophy (31,32). Additionally, Rho proteins may regulate the hypertrophic process by activating downstream signaling molecules such as mitogen-activated protein kinases (33). Additional work examining nonhypercholesterolemic transgenic rabbit models of hypertrophic cardiomyopathy demonstrated that simvastatin administration was associated with regression of cardiac hypertrophy and improvement of LV filling pressures (34). If lipid-lowering therapy has been shown to alter cardiac phenotypes, it is possible that the reverse effect may be true with increased cholesterol exposure.

A further aspect of this study is heightening the importance of raised serum triglycerides as a cardiovascular risk factor. Despite previous contention, triglycerides have emerged as a recognized causal risk factor (7). Current U.S. guidelines recommend intervention when triglycerides are >150 mg/dl (>1.7 mmol/l) (35), and European guidelines recommend the use of pharmacotherapy when triglycerides are >200 mg/dl (2.3 mmol/l) in high-risk patients and when lifestyle measures have failed (36). By way of illustration, 40% of our cohort had a serum triglyceride measurement of >150 mg/dl (1.7 mmol/l). Along with the recent data published by the REDUCE-IT (Reduction of Cardiovascular Events With Icosapent Ethyl-Intervention Trial) investigators (37,38), this study provides further evidence of the importance of triglycerides as a cardiovascular risk factor and, perhaps, will help in establishing a role for triglyceride reduction in a broader group of patients. In contrast, our findings of a lack of association with any LV remodeling parameter agree with the current narrative of HDL cholesterol not being associated with cardiovascular outcomes.

### Observational Analysis Versus Mr

A particularly interesting feature of this study is the discordance between the results produced from observational analysis compared to those derived from an MR approach. As examples, after adjusting for potential confounders, LDL cholesterol was shown to be observationally associated with significantly lower EDV and LV mass. However, the directionality of association was reversed in 1-sample and 2-sample MR. Moreover, observationally HDL cholesterol was associated with higher EDV and mass, whereas no significant association was demonstrated using MR. The MR approach has gained much traction because of its ability to permit experimental analysis free from the biases common to observational approaches. The results outlined are tacit in highlighting the limitations of observational methods. Of particular note are the observational results for LDL cholesterol, which prima facie suggest higher serum concentrations to be associated with ameliorative changes in the LV. This is in contrast to previous cross-sectional studies that have suggested adverse remodeling changes in association with non-HDL cholesterol and total cholesterol, respectively (39,40). That this study, particularly with its large sample size (N = 17,311), would deliver contrasting results in terms of both the previous literature and biological expectation is surprising. However, it may be instructive in characterizing a further challenge as biobank-based research becomes more common. As the degree of phenotyping undertaken by biobanks becomes more extensive, the temporal gap between different assessments will grow. For example, in this study, biochemistry samples for lipid quantification were drawn between 2006 and 2010, whereas CMR examinations have taken place since 2015. The observational analysis, therefore, is not strictly cross-sectional, and it is possible that the LDL cholesterol results were confounded by modulating factors that occurred between the 2 timepoints. One particular and relevant confounding intervention would be the introduction/continuation of statin therapy during the period before CMR examination. Although it was reassuring that examination of the relationship of measured LDL and genetically determined LDL demonstrated that statin use was consistently associated with relatively lower phenotypic LDL across the genetic LDL risk score range, a natural extension of this study would have been to investigate whether statins conferred any beneficial effect on LV parameters. Our examination of the effect modification by statin therapy on the association between the LDL GRS and LV parameters did not yield any significant results. However, a significant limitation is that data regarding commencement, duration, dosage, and dosage change of pharmacologic therapy (statins included) is not available in the UK Biobank.

### Study Strengths And Limitations

There are a number of strengths to this work—to our knowledge, the first to investigate the potentially causal relationship between routinely measured lipid fractions and prognostically important LV parameters. First, the effect estimates for building the genetic risk scores for LDL cholesterol, HDL cholesterol, and triglycerides were taken from an independent dataset (GLGC), which is one of the largest of its kind and has helped avoid circular inferences or overestimation in our results. Second, this study has sufficient sample size as confirmed by power calculations performed a priori to its commencement. Finally, both 1-sample and 2-sample MR have been performed, providing an additional level of confidence concerning the results provided.

In addition to the lack of pharmacotherapy data already explained, although supporting data providing mechanistic insights have been outlined, this study is unable to determine the specific molecular mechanism(s) for the potentially causal relationship between LDL cholesterol and triglycerides and alterations in LV parameters, although it is hoped that the findings presented may prompt further basic science investigations. Additional limitations mostly pertain to the MR technique. It is acknowledged that MR assumptions of independence and exclusion restriction cannot be fully tested, nor can residual horizontal pleiotropy be fully ruled out. However, as described, the MR-Egger intercepts did not deviate significantly from the origin. Bidirectional MR was not performed to determine whether LV genetic risk scores are causally associated with alterations in lipid measurements; this was because of the limited number of significant genome-wide variants for LV parameters. Nevertheless, MR-Steiger results suggested that assumptions of causal directionality were accurate. Finally, our study was restricted to Europeans because of the limited number of non-European participants in our CMR data. Thus, the insights gained cannot be extended to individuals of other ancestries. This limitation is likely to be over-come in the near future by the ongoing UK Biobank CMR study, with a target sample size of 100,000, as well as through collaboration with other maturing national biobanks, which will increase available data for individuals of other ethnicities.

## Conclusions

By performing MR, this study investigated the association between lipids and CMR parameters. It provides evidence that exposure to higher levels of LDL cholesterol and triglycerides are associated with changes in the LV known to portend an adverse prognosis. It improves our understanding of serum lipids as a risk factor for cardiovascular disease by demonstrating evidence of direct impact on cardiac structure and function.

## Abbreviations And Acronyms

BMI: body mass index
BSA: body surface area
CI: confidence interval
CMR: cardiovascular magnetic resonance
EDV: end-diastolic volume
EF: ejection fraction
GLGC: Global Lipids Genetic Consortium
GRS: genetic risk score
Hb: hemoglobin
HDL: high-density lipoprotein
IVW: inverse-variance weighted
LDL: low-density lipoprotein
LV: left ventricle
MR: Mendelian randomization

## Supplementary Material

Supplemental Tables 1-13 and Supplemental Figures 1-5

Appendix

## Figures and Tables

**Central Illustration:**
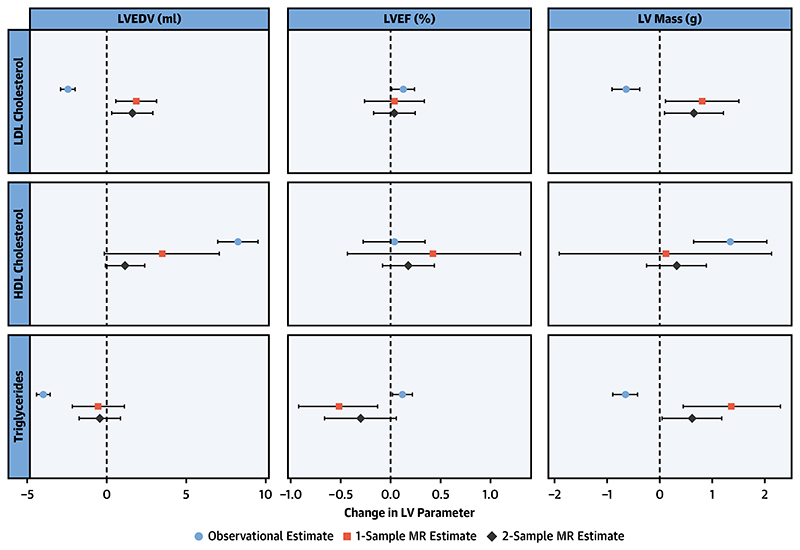
Mendelian Randomization Analysis Demonstrates a Causal Association Between Increased Low-Density Lipoprotein Cholesterol and Higher Left Ventricular End-Diastolic Volume and Left Ventricular Mass, and Triglycerides With Higher Left Ventricular Mass There is a potentially causal association between increased LDL cholesterol and higher LV end-diastolic volume and LV mass, as well as an association of triglycerides with higher LV mass, whereas HDL cholesterol does not result in any significant alterations in LV structure and function. Observational data are presented as change in LV parameter per 39 mg/dl (1 mmol/l) in LDL and HDL cholesterol and 89 mg/dl in triglycerides. One-sample MR data are presented as change in LV parameter per 39 mg/dl (1 mmol/l) for LDL and HDL cholesterol and 89 mg/dl for triglyceride increase in lifetime lipid parameter exposure. For 2-sample MR, data are presented as the change in LV parameter per 1 SD increase in LDL cholesterol (34 mg/dl [0.87 mmol/l]), HDL cholesterol (15 mg/dl [0.38 mmol/l]), and triglycerides (90 mg/dl [1.02 mmol/l]), respectively. HDL = high-density lipoprotein; LDL = low-density lipoprotein; LV = left ventricle; LVEDV = left ventricular end-diastolic volume; LVEF = left ventricular ejection fraction; MR = Mendelian randomization.

**Table 1:** Demographic Data

Percentile Group by Serum LHL Cholesterol Concentration						
	0%-50% (n = 215,845)	51%-75% (n = 109,763)	76%-90% (n = 66,350)	91%-95% (n = 22,204)	96%-100% (n = 21,902)	p Value for Trend
LDL cholesterol, mg/dl	115 ± 20	146 ± 19	165 ± 22	181 ± 25	200 ± 33	<0.001
HDL cholesterol, mg/dl	56 ± 15	56 ± 14	56 ± 13	57 ± 13	57 ± 13	<0.001
Triglycerides, mg/dl	132 ± 82	164 ± 89	181 ± 93	195 ± 96	210 ± 101	<0.001
Age, yrs	55.5 ± 8.4	57.6 ± 7.6	58.1 ± 7.3	58.4 ± 7.1	59.0 ± 6.9	<0.001
Male	99,700 (46.2)	51,742 (47.1)	29,740 (44.8)	9,482 (42.7)	8,721 (39.8)	<0.001
White	215,845 (100.0)	109,763 (100.0)	66,350 (100.0)	22,204 (100.0)	21,902 (100.0)	−
BMI, kg/m^2^	26.1 (23.5-29.3)	27.0 (24.5-30.1)	27.4 (24.9-30.4)	27.5 (25.1-30.3)	27.7 (25.3-30.7)	<0.001
Systolic BP, mm Hg	133 (123-147)	138 (127-151)	140 (128-152)	141 (129-154)	142 (130-155)	<0.001
On lipid-lowering medication	31,602 (14.6)	19,081 (17.4)	13,273 (20.0)	5,286 (23.8)	8,411 (38.4)	<0.001
On antihypertensive medications	47,173 (21.9)	25,262 (23.0)	15,148 (22.8)	5,071 (22.8)	5,691 (26.0)	<0.001
Diabetes mellitus	13,365 (6.2)	4,424 (4.0)	2,267 (3.4)	736 (3.3)	983 (4.5)	<0.001
HbA1c, mmol/mol	34.6 (32.1-37.3)	35.3 (33.0-37.9)	35.7 (33.5-38.1)	36.1 (33.8-38.5)	36.4 (34.1-38.9)	<0.001
CMR parameters (n = 17,311)						
LVEDV, ml	146 (126-171)	144 (123-170)	142 (122-167)	141 (120-166)	139 (119-161)	<0.001
LVESV, ml	59 (49-72)	58 (47-72)	57 (46-70)	56 (45-68)	55 (44-68)	<0.001
LVEF, %	59 (55-63)	59 (56-63)	60 (56-64)	60 (56-64)	60 (56-63)	<0.001
LV mass, g	82 (69-101)	84 (69-103)	85 (70-103)	83 (69-101)	86 (69-102)	0.015

Values are mean ± SD, n (%), or median (interquartile range).

BMI = body mass index; BP = blood pressure; CMR = cardiovascular magnetic resonance; Hb = hemoglobin; HDL = high-density lipoprotein; LDL = low-density lipoprotein; LV = left ventricular; LVEDV = left ventricular end-diastolic volume; LVEF = left ventricular ejection fraction; LVESV = left ventricular end-systolic volume.

**Table 2:** Effect of Phenotypic and Genetically Determined Lipid Levels on LV Parameters

		Observational			MR				
Lipid Parameter	Phenotype	Effect Size	95% CI	p Value	Effect Size	95% CI	p Value	Durbin-Wu-Hausman p Value	*F* Statistic
LDL cholesterol	LVEDV, ml	-2.44	-2.91 to -1.97	< 0.0001	1.85	0.59 to 3.14	0.004	< 0.0001	2,492.0
	LVEF, %	0.13	0.01 to 0.24	0.03	0.04	-0.26 to 0.34	0.80	0.22	2,492.0
	LV mass, g	-0.64	-0.90 to -0.38	< 0.0001	0.81	0.11 to 1.51	0.023	0.0005	2,492.0
HDL cholesterol	LVEDV, ml	8.27	7.00 to 9.53	< 0.0001	3.48	-0.15 to 7.08	0.056	< 0.0001	1,811.2
	LVEF, %	0.04	-0.27 to 0.35	0.806	0.43	-0.43 to 1.30	0.312	0.403	1,811.2
	LV mass, g	1.34	0.64 to 2.04	0.0002	0.11	-1.91 to 2.13	0.914	0.076	1,811.2
Triglycerides	LVEDV, ml	-3.98	-4.40 to -3.55	< 0.0001	-0.54	-2.17 to 1.12	0.517	< 0.0001	925.1
	LVEF, %	0.12	0.02 to 0.22	0.024	-0.52	-0.92 to -0.13	0.011	0.0003	925.1
	LV mass, g	-0.65	-0.89 to -0.42	< 0.0001	1.37	0.45 to 2.30	0.004	0.0002	925.1

Observational data are adjusted for age at recruitment, sex, BMI, body surface area, systolic blood pressure adjusted for antihypertensive medication use, physical activity, smoking status, HbA1c, and presenceofcardiovasculardisease; data are presented forchange in LV parameter per39 mg/dl(1 mmol/l) in LDL and HDL cholesterol and 89 mg/dl in triglycerides. One-sample MRdata are adjusted forage, sex, body surface area, and the first 5 principal components, and data are presented as change in LV parameter per 39 mg/dl (1 mmol/l) for LDL and HDL cholesterol and 89 mg/dl for triglyceride increase in lifetime lipid parameter exposure.

CI = confidence interval; MR = Mendelian randomization; other abbreviations as in Table 1.

**Table 3:** 2-Sample MR Analysis Using Summary-Level Data

		IVW				
Lipid Parameter	Phenotype	Effect Size	95% CI	p Value	Egger Intercept	Egger Intercept p Value
LDL cholesterol	LVEDV, ml	1.62	0.32 to 2.91	0.014	-0.023	0.655
	LVEF, %	0.04	-0.17 to 0.25	0.705	-0.007	0.490
	LV mass, g	0.66	0.10 to 1.22	0.021	0.024	0.368
HDL cholesterol	LVEDV, ml	1.16	-0.07 to 2.39	0.065	0.013	0.820
	LVEF, %	0.18	-0.08 to 0.44	0.184	-0.003	0.812
	LV mass, g	0.32	-0.26 to 0.89	0.279	-0.029	0.296
Triglycerides	LVEDV, ml	-0.43	-1.73 to 0.86	0.512	-0.039	0.504
	LVEF, %	-0.30	-0.66 to 0.06	0.106	-0.002	0.889
	LV mass, g	0.61	0.04 to 1.18	0.036	0.034	0.188

For2-sample MR, the change in LV parameter reflects an increase per 34 mg/dl (0.87 mmol/l), 15 mg/dl (0.38 mmol/l), and 90 mg/dl (1.02 mmol/l) increase in LDL cholesterol, HDL cholesterol, and triglycerides, respectively.

IVW = inverse-variance weighted; other abbreviations as in Tables 1 and 2.

## Perspectives

### Competency In Medical Knowledge

Although the results of MR analyses are, to some extent, discordant with observational data, elevated LDL-cholesterol levels are causally associated with increased LV mass and EDV. Triglycerides are associated with higher LV mass, whereas HDL cholesterol is not associated with these LV indices.

### Translational Outlook

Further research is needed to understand the direct, nonatherogenic effects of blood lipids on myocardial structure and function.
