# Supplementary material for: The Effect of Blood Lipids on the Left Ventricle: A Mendelian Randomization Study
Source: J Am Coll Cardiol. Author manuscript; Available in PMC 2022 Aug 6. (PMC7613249; doi:10.1016/j.jacc.2020.09.583)
Supplement: Supplemental Tables 1-13 and Supplemental Figures 1-5 [file EMS151235-supplement-Supplemental_Tables_1_13_and_Supplemental_Figures_1_5.docx]

**Supplementary Material**

**Supplementary Tables**

Supplementary Table 1: 101 variants included in the genetic risk score for LDL cholesterol

Supplementary Table 2: 125 variants included in the genetic risk score for HDL cholesterol

Supplementary Table 3: 73 variants included in the genetic risk score for triglycerides

Supplementary Table 4. One-sample MR with a single instrument and additional adjustment for all three lipid instruments

Supplementary Table 5. One-sample MR adjusted for all potential confounders

Supplementary Table 6: Associations between LV parameters and lipid parameter with phenotypic and genetically-determined lipid level included as covariates Supplementary Table 7: One-sample MR results using restricted list of variants in GRS

Supplementary Table 8: Two-sample single instrument and multivariable MR analysis using summary level data

Supplementary Table 9: Two-sample MR with MR-Egger, weighted median and weighted mode methods

Supplementary Table 10: MR pleiotropy residual sum and outlier (MR-PRESSO) analysis for horizontal pleiotropy

Supplementary Table 11: MR-Steiger analysis

Supplementary Table 12: The variance of phenotypic LV parameters explained by observed lipid measurements and lipid genetic risk scores

Supplementary Table 13. Interaction analysis of the statin therapy on the relationships between the genetic risk score for LDL cholesterol and LV parameters

**Supplementary Figures**

Supplementary Figure 1: Power analysis for one-sample MR

Supplementary Figure 2. Correlation between lipid genetic risk scores assessed by Pearson’s test

Supplementary Figure 3. Forest plots for two-sample MR analysis of LDL cholesterol

Supplementary Figure 4. Forest plots for two-sample MR analysis of HDL cholesterol

Supplementary Figure 5. Forest plots for two-sample MR analysis of triglycerides

| **Supplementary Table 1: 101 variants included in the genetic risk score for LDL cholesterol** | | | | | | |
| --- | --- | --- | --- | --- | --- | --- |
| **Variant ID** | **Effect allele** | **Other allele** | **Effect allele frequency** | **Beta** | **SE** | **P** |
| rs2419604 | A | G | 0.3179 | 0.0302 | 0.004 | 7.49E-14 |
| rs413380 | C | T | 0.9657 | 0.0861 | 0.0098 | 7.62E-17 |
| rs4970834 | C | T | 0.8127 | 0.1503 | 0.0047 | 1.00E-200 |
| rs9804646 | C | T | 0.91029 | 0.0454 | 0.007 | 8.60E-11 |
| rs10893499 | A | G | 0.1438 | 0.0521 | 0.0053 | 3.86E-21 |
| rs10832962 | T | C | 0.719 | 0.032 | 0.004 | 6.62E-14 |
| rs267733 | A | G | 0.8628 | 0.0331 | 0.0053 | 5.29E-09 |
| rs174583 | C | T | 0.6253 | 0.0522 | 0.0038 | 7.00E-41 |
| rs3184504 | C | T | 0.5343 | 0.0268 | 0.0038 | 4.20E-12 |
| rs1169288 | C | A | 0.3338 | 0.0375 | 0.004 | 6.45E-21 |
| rs2642438 | G | A | 0.7454 | 0.0352 | 0.0042 | 7.32E-16 |
| rs2587534 | A | G | 0.5277 | 0.0391 | 0.0037 | 8.06E-25 |
| rs10903129 | G | A | 0.5369 | 0.0328 | 0.0037 | 3.03E-17 |
| rs12748152 | T | C | 0.07124 | 0.0499 | 0.0066 | 3.21E-12 |
| rs4942486 | T | C | 0.4617 | 0.0243 | 0.0037 | 2.26E-11 |
| rs8017377 | A | G | 0.4591 | 0.0303 | 0.0038 | 2.52E-15 |
| rs11206508 | A | G | 0.1319 | 0.0434 | 0.0055 | 2.26E-14 |
| rs17111503 | G | A | 0.2414 | 0.0662 | 0.0045 | 1.39E-45 |
| rs11591147 | G | T | 0.98285 | 0.497 | 0.018 | 8.60E-143 |
| rs630431 | A | G | 0.6913 | 0.0351 | 0.0042 | 7.73E-17 |
| rs11583974 | A | G | 0.03034 | 0.0646 | 0.0117 | 3.95E-09 |
| rs2647281 | G | A | 0.05541 | 0.0589 | 0.0095 | 2.27E-09 |
| rs207150 | C | T | 0.91821 | 0.0472 | 0.0065 | 2.00E-12 |
| rs11485618 | A | G | 0.6913 | 0.05 | 0.0039 | 3.73E-33 |
| rs247616 | C | T | 0.7071 | 0.0547 | 0.0041 | 2.57E-37 |
| rs2000999 | A | G | 0.1847 | 0.065 | 0.0046 | 4.22E-41 |
| rs6504872 | T | C | 0.4723 | 0.0274 | 0.0037 | 3.48E-13 |
| rs1801689 | C | A | 0.03694 | 0.1028 | 0.0139 | 9.81E-12 |
| rs2886232 | T | C | 0.1201 | 0.0451 | 0.0064 | 3.88E-11 |
| rs314253 | T | C | 0.6649 | 0.0242 | 0.0038 | 3.44E-10 |
| rs11669133 | A | G | 0.04222 | 0.0501 | 0.0098 | 4.80E-08 |
| rs6511720 | G | T | 0.90237 | 0.2209 | 0.0061 | 1.00E-200 |
| rs688 | T | C | 0.4472 | 0.054 | 0.0037 | 1.01E-43 |
| rs6511727 | T | G | 0.3852 | 0.0266 | 0.0038 | 1.84E-11 |
| rs376642 | C | T | 0.715 | 0.0233 | 0.004 | 4.67E-10 |
| rs10401969 | T | C | 0.92876 | 0.1184 | 0.0072 | 2.65E-54 |
| rs4970712 | C | A | 0.8061 | 0.0339 | 0.0044 | 2.46E-13 |
| rs17800760 | G | A | 0.8681 | 0.0513 | 0.0053 | 8.87E-22 |
| rs10460181 | A | G | 0.8127 | 0.0536 | 0.0046 | 2.25E-28 |
| rs1531517 | G | A | 0.94855 | 0.2202 | 0.008 | 9.50E-163 |
| rs7254892 | G | A | 0.96834 | 0.4853 | 0.0119 | 1.00E-200 |
| rs2075650 | G | A | 0.1266 | 0.1767 | 0.0055 | 1.00E-200 |
| rs75687619 | T | G | 0.02375 | 0.1735 | 0.0161 | 8.05E-24 |
| rs2287019 | C | T | 0.81 | 0.0283 | 0.0048 | 8.36E-09 |
| rs492602 | G | A | 0.4301 | 0.0293 | 0.0039 | 9.42E-14 |
| rs364585 | G | A | 0.6332 | 0.0249 | 0.0038 | 4.28E-10 |
| rs2328223 | C | A | 0.2493 | 0.0299 | 0.005 | 5.63E-09 |
| rs7264396 | C | T | 0.781 | 0.0246 | 0.0045 | 4.41E-08 |
| rs6016381 | T | C | 0.6398 | 0.0363 | 0.0038 | 6.85E-20 |
| rs6065311 | C | T | 0.4604 | 0.0417 | 0.0036 | 1.66E-30 |
| rs1800961 | C | T | 0.9657 | 0.0685 | 0.0106 | 6.03E-10 |
| rs10490626 | G | A | 0.92084 | 0.0508 | 0.0069 | 1.70E-12 |
| rs2030746 | T | C | 0.3984 | 0.0214 | 0.0038 | 8.61E-09 |
| rs16831243 | T | C | 0.1807 | 0.0378 | 0.0055 | 9.06E-12 |
| rs10195252 | T | C | 0.5818 | 0.0238 | 0.0039 | 3.81E-08 |
| rs492399 | G | A | 0.03562 | 0.0629 | 0.0102 | 1.23E-09 |
| rs13414987 | A | C | 0.2177 | 0.0308 | 0.0043 | 9.94E-12 |
| rs1367117 | A | G | 0.2876 | 0.1186 | 0.004 | 9.50E-183 |
| rs12471982 | C | A | 0.1359 | 0.0365 | 0.0054 | 3.93E-11 |
| rs520861 | G | A | 0.7071 | 0.0843 | 0.0042 | 1.78E-84 |
| rs1250229 | C | T | 0.7889 | 0.0243 | 0.0042 | 3.13E-08 |
| rs5763662 | T | C | 0.02507 | 0.0767 | 0.0121 | 1.19E-08 |
| rs11563251 | T | C | 0.1253 | 0.0345 | 0.0062 | 4.50E-08 |
| rs4253776 | G | A | 0.124 | 0.0311 | 0.0059 | 3.35E-08 |
| rs780093 | T | C | 0.4129 | 0.0223 | 0.0037 | 2.36E-08 |
| rs1025447 | C | T | 0.1583 | 0.0418 | 0.0048 | 3.78E-16 |
| rs6544713 | T | C | 0.2942 | 0.0806 | 0.0041 | 4.84E-83 |
| rs6709904 | A | G | 0.8865 | 0.055 | 0.0085 | 4.58E-10 |
| rs2710642 | A | G | 0.6187 | 0.0239 | 0.0038 | 6.09E-09 |
| rs9875338 | G | A | 0.6121 | 0.027 | 0.0037 | 2.21E-11 |
| rs17404153 | G | T | 0.8562 | 0.0336 | 0.0054 | 1.83E-09 |
| rs7640978 | C | T | 0.8945 | 0.0392 | 0.0069 | 9.84E-09 |
| rs6818397 | T | G | 0.4129 | 0.0224 | 0.004 | 1.68E-08 |
| rs4530754 | A | G | 0.5818 | 0.0275 | 0.0036 | 3.58E-12 |
| rs6882076 | C | T | 0.6662 | 0.0456 | 0.0038 | 3.31E-31 |
| rs12916 | C | T | 0.4314 | 0.0733 | 0.0038 | 7.79E-78 |
| rs6909746 | C | T | 0.6082 | 0.0263 | 0.0037 | 7.86E-11 |
| rs1564348 | C | T | 0.1451 | 0.0481 | 0.005 | 2.76E-21 |
| rs3125055 | A | T | 0.1398 | 0.0468 | 0.0055 | 5.92E-16 |
| rs1510226 | C | T | 0.01319 | 0.1409 | 0.0214 | 1.71E-10 |
| rs7770628 | C | T | 0.4485 | 0.0258 | 0.0037 | 3.17E-11 |
| rs3757354 | C | T | 0.7902 | 0.0382 | 0.0044 | 2.09E-17 |
| rs13206249 | G | A | 0.7836 | 0.0378 | 0.0062 | 4.53E-08 |
| rs1800562 | G | A | 0.95383 | 0.0615 | 0.008 | 8.25E-14 |
| rs2247056 | C | T | 0.7823 | 0.0248 | 0.0043 | 1.42E-08 |
| rs10947332 | A | G | 0.1319 | 0.0504 | 0.0056 | 6.97E-18 |
| rs12670798 | C | T | 0.2243 | 0.0344 | 0.0043 | 4.81E-14 |
| rs4722551 | C | T | 0.1702 | 0.0391 | 0.0049 | 3.95E-14 |
| rs2073547 | G | A | 0.1939 | 0.0485 | 0.0049 | 1.92E-21 |
| rs2737252 | G | A | 0.7441 | 0.0314 | 0.0041 | 7.04E-14 |
| rs2954029 | A | T | 0.5317 | 0.0564 | 0.0036 | 2.10E-50 |
| rs7832643 | T | G | 0.405 | 0.0339 | 0.0038 | 2.67E-17 |
| rs10102164 | A | G | 0.1741 | 0.0316 | 0.0045 | 3.74E-11 |
| rs13277801 | C | T | 0.347 | 0.0338 | 0.0038 | 3.99E-17 |
| rs9987289 | G | A | 0.9248 | 0.0714 | 0.0066 | 8.53E-24 |
| rs1883025 | C | T | 0.7573 | 0.0296 | 0.0044 | 6.14E-11 |
| rs8176722 | C | A | 0.8892 | 0.0473 | 0.006 | 1.85E-14 |
| rs579459 | C | T | 0.215 | 0.0665 | 0.0045 | 2.42E-44 |
| rs3780181 | A | G | 0.94723 | 0.0445 | 0.0074 | 1.76E-09 |
| rs519113 | C | G | 0.786 | 0.0971 | 0.0066 | 1.61E-49 |
| rs964184 | G | C | 0.162 | 0.0855 | 0.0078 | 2.01E-26 |

| **Supplementary Table 2: 125 variants included in the genetic risk score for HDL cholesterol** | | | | | | |
| --- | --- | --- | --- | --- | --- | --- |
| **Variant ID** | **Effect allele** | **Other allele** | **Effect allele frequency** | **Beta** | **SE** | **P** |
| rs2250802 | G | A | 0.3193 | 0.034 | 0.0038 | 2.02E-17 |
| rs2148489 | T | C | 0.7757 | 0.0283 | 0.0041 | 1.41E-10 |
| rs970548 | C | A | 0.277 | 0.0258 | 0.0039 | 1.71E-10 |
| rs10761771 | C | T | 0.467 | 0.0198 | 0.0034 | 4.12E-09 |
| rs12740374 | T | G | 0.2124 | 0.0343 | 0.0041 | 1.69E-15 |
| rs333947 | G | A | 0.8536 | 0.0296 | 0.0047 | 3.17E-09 |
| rs7943309 | A | G | 0.03958 | 0.0865 | 0.0088 | 1.18E-20 |
| rs7117842 | C | T | 0.3892 | 0.0272 | 0.0035 | 1.06E-14 |
| rs17135399 | A | G | 0.93404 | 0.0483 | 0.0077 | 4.26E-09 |
| rs7128597 | C | A | 0.1715 | 0.0398 | 0.0065 | 1.82E-08 |
| rs3847502 | A | C | 0.314 | 0.048 | 0.0036 | 3.31E-38 |
| rs4752894 | G | A | 0.3984 | 0.0206 | 0.0035 | 1.89E-09 |
| rs12145743 | G | T | 0.3311 | 0.0203 | 0.0036 | 1.80E-08 |
| rs102275 | T | C | 0.628 | 0.0391 | 0.0035 | 6.40E-28 |
| rs12801636 | A | G | 0.2243 | 0.0235 | 0.0042 | 3.15E-08 |
| rs499974 | C | A | 0.8245 | 0.0263 | 0.0044 | 1.12E-08 |
| rs4650994 | G | A | 0.5172 | 0.021 | 0.0034 | 6.70E-09 |
| rs1689797 | C | A | 0.6979 | 0.0358 | 0.0036 | 2.85E-21 |
| rs2241210 | G | A | 0.5528 | 0.0332 | 0.0035 | 2.49E-20 |
| rs653178 | T | C | 0.5317 | 0.0263 | 0.0035 | 1.06E-12 |
| rs2454722 | G | A | 0.1451 | 0.0351 | 0.0044 | 3.31E-14 |
| rs11057397 | T | C | 0.3668 | 0.0282 | 0.0036 | 6.77E-14 |
| rs863750 | C | T | 0.4195 | 0.0264 | 0.0035 | 4.71E-13 |
| rs838876 | A | G | 0.3259 | 0.0493 | 0.0039 | 7.33E-33 |
| rs7306660 | G | A | 0.6306 | 0.0345 | 0.0036 | 3.34E-19 |
| rs7298751 | G | A | 0.1187 | 0.0434 | 0.0052 | 2.46E-16 |
| rs2642438 | G | A | 0.7454 | 0.0303 | 0.0039 | 7.78E-14 |
| rs11045163 | G | A | 0.4063 | 0.0217 | 0.0035 | 3.20E-09 |
| rs4846914 | A | G | 0.5844 | 0.0479 | 0.0034 | 3.51E-41 |
| rs3741414 | T | C | 0.1913 | 0.0296 | 0.004 | 6.10E-14 |
| rs12748152 | C | T | 0.92876 | 0.0506 | 0.0062 | 9.74E-16 |
| rs4660293 | A | G | 0.7639 | 0.0353 | 0.004 | 2.86E-18 |
| rs4983559 | G | A | 0.3773 | 0.0197 | 0.0036 | 9.57E-09 |
| rs492571 | T | C | 0.95778 | 0.0663 | 0.009 | 1.27E-12 |
| rs2899624 | A | G | 0.8456 | 0.0714 | 0.0049 | 1.39E-40 |
| rs185481 | C | T | 0.529 | 0.0366 | 0.0035 | 1.40E-23 |
| rs16940147 | A | G | 0.04617 | 0.0514 | 0.008 | 2.45E-10 |
| rs10468017 | T | C | 0.2757 | 0.1179 | 0.0038 | 1.20E-188 |
| rs1077834 | C | T | 0.2111 | 0.1253 | 0.0041 | 7.80E-180 |
| rs424346 | T | C | 0.04881 | 0.0679 | 0.0113 | 4.84E-08 |
| rs1007076 | C | T | 0.7296 | 0.0247 | 0.0041 | 4.43E-09 |
| rs1121980 | G | A | 0.5528 | 0.0196 | 0.0034 | 6.79E-09 |
| rs3790106 | C | G | 0.81 | 0.0374 | 0.0052 | 3.27E-11 |
| rs4784659 | T | C | 0.1847 | 0.0274 | 0.0049 | 1.02E-08 |
| rs13336936 | T | C | 0.03562 | 0.0717 | 0.0104 | 6.98E-11 |
| rs7193072 | A | G | 0.2375 | 0.0498 | 0.0038 | 1.40E-34 |
| rs1138429 | A | T | 0.90237 | 0.1156 | 0.0065 | 9.52E-66 |
| rs9989419 | G | A | 0.595 | 0.1473 | 0.0036 | 1.00E-200 |
| rs4783961 | A | G | 0.4855 | 0.0997 | 0.0036 | 5.70E-162 |
| rs289745 | A | C | 0.6029 | 0.0276 | 0.0041 | 2.28E-20 |
| rs291040 | T | C | 0.6623 | 0.0305 | 0.0037 | 8.21E-17 |
| rs16942887 | A | G | 0.1332 | 0.0831 | 0.0051 | 8.28E-54 |
| rs4986970 | A | T | 0.96702 | 0.0792 | 0.0099 | 1.09E-15 |
| rs2925979 | C | T | 0.7045 | 0.0351 | 0.0037 | 1.32E-19 |
| rs1877031 | A | G | 0.6755 | 0.0336 | 0.0036 | 1.20E-19 |
| rs4148005 | T | G | 0.7005 | 0.0283 | 0.0036 | 5.74E-14 |
| rs4969178 | G | A | 0.6266 | 0.0263 | 0.0035 | 1.53E-12 |
| rs8093249 | A | G | 0.8404 | 0.0384 | 0.0051 | 1.80E-13 |
| rs9955201 | A | G | 0.06069 | 0.0638 | 0.0081 | 2.40E-14 |
| rs4939883 | C | T | 0.8193 | 0.0799 | 0.0045 | 1.80E-66 |
| rs9951669 | G | A | 0.2177 | 0.0408 | 0.0042 | 3.01E-21 |
| rs6567160 | T | C | 0.7691 | 0.0257 | 0.0041 | 2.92E-09 |
| rs737337 | T | C | 0.9314 | 0.0565 | 0.0061 | 4.56E-17 |
| rs12133576 | A | G | 0.3549 | 0.0243 | 0.0035 | 6.15E-11 |
| rs731839 | A | G | 0.6583 | 0.022 | 0.0037 | 3.44E-09 |
| rs2075650 | A | G | 0.8734 | 0.0554 | 0.0051 | 9.72E-26 |
| rs77301115 | G | A | 0.97361 | 0.0972 | 0.0157 | 1.03E-08 |
| rs7412 | T | C | 0.06596 | 0.0978 | 0.0097 | 4.44E-19 |
| rs5167 | G | T | 0.3694 | 0.032 | 0.0037 | 4.88E-16 |
| rs17695224 | G | A | 0.7612 | 0.029 | 0.0039 | 2.42E-13 |
| rs103294 | T | C | 0.186 | 0.0523 | 0.0044 | 4.00E-30 |
| rs2278236 | A | G | 0.5435 | 0.0331 | 0.0035 | 3.19E-18 |
| rs3111576 | T | C | 0.1372 | 0.0448 | 0.0054 | 1.20E-14 |
| rs1800961 | C | T | 0.9657 | 0.127 | 0.0099 | 1.64E-34 |
| rs4465830 | A | G | 0.7982 | 0.0597 | 0.0044 | 5.18E-40 |
| rs17380117 | A | G | 0.8087 | 0.0253 | 0.0042 | 3.85E-09 |
| rs7607980 | C | T | 0.1491 | 0.0447 | 0.0052 | 1.81E-15 |
| rs676210 | A | G | 0.2309 | 0.066 | 0.004 | 2.35E-54 |
| rs1047891 | C | A | 0.6979 | 0.0269 | 0.0039 | 8.73E-10 |
| rs181360 | T | G | 0.8008 | 0.0376 | 0.0042 | 9.24E-18 |
| rs1515110 | G | T | 0.3813 | 0.0323 | 0.0035 | 8.04E-18 |
| rs2606736 | C | T | 0.3945 | 0.0246 | 0.0043 | 4.80E-08 |
| rs6805251 | T | C | 0.3813 | 0.02 | 0.0035 | 1.33E-08 |
| rs13076253 | A | C | 0.8522 | 0.0283 | 0.0048 | 4.96E-09 |
| rs687339 | C | T | 0.2335 | 0.0316 | 0.0042 | 7.11E-13 |
| rs2290547 | G | A | 0.7889 | 0.0297 | 0.0046 | 3.69E-09 |
| rs2013208 | T | C | 0.5053 | 0.0254 | 0.0036 | 8.92E-12 |
| rs13326165 | A | G | 0.1873 | 0.0289 | 0.0043 | 9.04E-11 |
| rs2602836 | A | G | 0.4274 | 0.0192 | 0.0034 | 4.96E-08 |
| rs13107325 | C | T | 0.92216 | 0.0708 | 0.0078 | 1.07E-15 |
| rs10019888 | A | G | 0.8364 | 0.027 | 0.0046 | 4.90E-08 |
| rs442177 | G | T | 0.4472 | 0.0215 | 0.0034 | 2.19E-09 |
| rs3822072 | G | A | 0.5119 | 0.0251 | 0.0034 | 4.06E-12 |
| rs6450176 | G | A | 0.7216 | 0.0254 | 0.0039 | 6.88E-10 |
| rs3936511 | A | G | 0.8311 | 0.0308 | 0.0046 | 2.96E-09 |
| rs1936800 | C | T | 0.5277 | 0.02 | 0.0034 | 3.06E-10 |
| rs3861397 | A | G | 0.6583 | 0.024 | 0.0036 | 8.40E-11 |
| rs9457931 | A | G | 0.9314 | 0.0552 | 0.0073 | 7.30E-13 |
| rs3823417 | A | G | 0.2322 | 0.0285 | 0.0042 | 2.07E-11 |
| rs715299 | T | G | 0.7441 | 0.024 | 0.0039 | 6.47E-09 |
| rs205262 | A | G | 0.7335 | 0.0283 | 0.0039 | 3.88E-13 |
| rs998584 | C | A | 0.4855 | 0.026 | 0.0038 | 2.27E-11 |
| rs11765979 | C | A | 0.4578 | 0.0412 | 0.0048 | 3.11E-17 |
| rs13225097 | A | G | 0.7678 | 0.0227 | 0.0039 | 4.33E-08 |
| rs17173637 | T | C | 0.90237 | 0.0363 | 0.0057 | 1.90E-08 |
| rs4142995 | G | T | 0.6161 | 0.0263 | 0.0037 | 9.37E-12 |
| rs4917014 | G | T | 0.3404 | 0.0222 | 0.0036 | 1.03E-08 |
| rs702485 | G | A | 0.4499 | 0.0243 | 0.0034 | 6.45E-12 |
| rs17145738 | T | C | 0.1174 | 0.0408 | 0.0053 | 4.95E-13 |
| rs7014168 | G | A | 0.7586 | 0.0267 | 0.0041 | 9.20E-10 |
| rs2293889 | G | T | 0.5871 | 0.0312 | 0.0035 | 4.27E-17 |
| rs10808546 | T | C | 0.4459 | 0.0409 | 0.0034 | 4.11E-30 |
| rs10087900 | G | A | 0.5607 | 0.0231 | 0.0036 | 2.17E-09 |
| rs7016529 | T | C | 0.98681 | 0.2186 | 0.0141 | 9.27E-45 |
| rs13702 | C | T | 0.3127 | 0.1058 | 0.0038 | 1.30E-160 |
| rs13265868 | A | G | 0.4604 | 0.0478 | 0.0035 | 6.10E-40 |
| rs16842 | T | C | 0.7467 | 0.03 | 0.0038 | 3.82E-14 |
| rs4240624 | A | G | 0.9248 | 0.0818 | 0.0058 | 1.32E-45 |
| rs2230808 | C | T | 0.7889 | 0.0385 | 0.004 | 1.59E-20 |
| rs2853579 | T | G | 0.1108 | 0.0499 | 0.0053 | 1.32E-19 |
| rs11789603 | T | C | 0.08971 | 0.06 | 0.006 | 3.70E-21 |
| rs1883025 | C | T | 0.7573 | 0.0698 | 0.0041 | 1.50E-65 |
| rs686030 | A | C | 0.8588 | 0.055 | 0.0049 | 4.29E-27 |
| rs964184 | C | G | 0.838 | 0.1065 | 0.0071 | 6.09E-48 |
| rs6589581 | T | A | 0.021 | 0.0845 | 0.0137 | 2.26E-09 |

| **Supplementary Table 3: 73 variants included in the genetic risk score for triglycerides** | | | | | | |
| --- | --- | --- | --- | --- | --- | --- |
| **Variant ID** | **Effect allele** | **Other allele** | **Effect allele frequency** | **Beta** | **SE** | **P** |
| rs2250802 | A | G | 0.6807 | 0.023 | 0.0037 | 1.21E-10 |
| rs1832007 | A | G | 0.8681 | 0.0327 | 0.0047 | 1.72E-12 |
| rs10761762 | T | C | 0.533 | 0.027 | 0.0033 | 1.06E-17 |
| rs2068888 | G | A | 0.5092 | 0.0241 | 0.0034 | 1.68E-11 |
| rs7350481 | T | C | 0.09763 | 0.2254 | 0.0066 | 1.00E-200 |
| rs2187126 | A | G | 0.94591 | 0.0543 | 0.0069 | 2.90E-15 |
| rs12294259 | T | C | 0.05937 | 0.219 | 0.0069 | 1.80E-200 |
| rs9804646 | C | T | 0.91029 | 0.0524 | 0.0064 | 2.82E-17 |
| rs5110 | A | C | 0.06464 | 0.156 | 0.0124 | 2.14E-34 |
| rs7943309 | G | A | 0.96042 | 0.0605 | 0.0087 | 1.16E-11 |
| rs10501321 | T | C | 0.686 | 0.0216 | 0.0035 | 1.41E-08 |
| rs174535 | C | T | 0.3628 | 0.047 | 0.0034 | 1.73E-41 |
| rs11057408 | G | T | 0.6372 | 0.0258 | 0.0035 | 2.05E-12 |
| rs1321257 | G | A | 0.4063 | 0.0402 | 0.0034 | 5.99E-31 |
| rs11613352 | C | T | 0.8087 | 0.028 | 0.0039 | 9.40E-14 |
| rs12748152 | T | C | 0.07124 | 0.0372 | 0.0059 | 1.10E-09 |
| rs17513135 | T | C | 0.2322 | 0.022 | 0.0039 | 1.63E-08 |
| rs16948098 | A | G | 0.0409 | 0.08 | 0.0089 | 4.84E-17 |
| rs10468017 | T | C | 0.2757 | 0.0379 | 0.0039 | 7.56E-21 |
| rs588136 | C | T | 0.2058 | 0.0495 | 0.0041 | 3.37E-30 |
| rs3198697 | C | T | 0.6174 | 0.0198 | 0.0034 | 2.21E-08 |
| rs4587594 | G | A | 0.69 | 0.0694 | 0.0035 | 3.50E-82 |
| rs749671 | G | A | 0.6055 | 0.0211 | 0.0034 | 6.11E-10 |
| rs9930333 | G | T | 0.4485 | 0.0208 | 0.0037 | 3.25E-08 |
| rs247616 | C | T | 0.7071 | 0.0393 | 0.0037 | 1.12E-25 |
| rs5880 | C | G | 0.05937 | 0.0475 | 0.0085 | 4.71E-08 |
| rs8077889 | C | A | 0.2441 | 0.0252 | 0.0042 | 9.88E-09 |
| rs117877390 | C | T | 0.9657 | 0.1099 | 0.0141 | 1.53E-09 |
| rs10401969 | T | C | 0.92876 | 0.121 | 0.0065 | 9.70E-70 |
| rs731839 | G | A | 0.3417 | 0.0224 | 0.0036 | 2.65E-09 |
| rs4803750 | G | A | 0.05541 | 0.0423 | 0.007 | 9.52E-09 |
| rs7254892 | A | G | 0.03166 | 0.1235 | 0.0106 | 1.40E-24 |
| rs439401 | C | T | 0.6201 | 0.0659 | 0.0038 | 1.42E-66 |
| rs3760627 | C | T | 0.4683 | 0.0189 | 0.0034 | 5.29E-09 |
| rs7248104 | G | A | 0.5831 | 0.0222 | 0.0034 | 5.05E-10 |
| rs4804311 | A | G | 0.8905 | 0.0392 | 0.006 | 1.49E-09 |
| rs6029143 | C | T | 0.94195 | 0.0388 | 0.0071 | 4.93E-08 |
| rs4810479 | C | T | 0.2876 | 0.0474 | 0.0038 | 2.07E-34 |
| rs6066141 | T | C | 0.7586 | 0.0297 | 0.0053 | 2.34E-08 |
| rs13389219 | C | T | 0.591 | 0.0271 | 0.0034 | 2.60E-15 |
| rs676210 | G | A | 0.7691 | 0.0733 | 0.0039 | 3.28E-71 |
| rs2972146 | T | G | 0.6227 | 0.0281 | 0.0034 | 2.97E-15 |
| rs3761445 | A | G | 0.6148 | 0.0232 | 0.0034 | 8.06E-12 |
| rs2304684 | T | C | 0.02507 | 0.086 | 0.0127 | 5.00E-11 |
| rs1260326 | T | C | 0.4129 | 0.1148 | 0.0034 | 1.00E-200 |
| rs11674085 | A | G | 0.2005 | 0.0251 | 0.0044 | 2.86E-08 |
| rs10440120 | C | A | 0.8325 | 0.0306 | 0.0044 | 5.34E-11 |
| rs645040 | T | G | 0.7691 | 0.0293 | 0.004 | 1.83E-12 |
| rs6831256 | G | A | 0.409 | 0.0258 | 0.0035 | 1.60E-12 |
| rs442177 | T | G | 0.5528 | 0.0309 | 0.0033 | 1.32E-18 |
| rs6882076 | C | T | 0.6662 | 0.0286 | 0.0035 | 1.51E-15 |
| rs9686661 | T | C | 0.1768 | 0.0379 | 0.0044 | 2.54E-16 |
| rs719726 | T | C | 0.529 | 0.0199 | 0.0035 | 2.49E-08 |
| rs634869 | T | C | 0.438 | 0.0272 | 0.0033 | 1.78E-14 |
| rs2665357 | C | A | 0.5092 | 0.0212 | 0.0033 | 8.33E-10 |
| rs2508015 | G | A | 0.6715 | 0.0252 | 0.0038 | 1.33E-10 |
| rs2247056 | C | T | 0.7823 | 0.0378 | 0.0039 | 3.86E-21 |
| rs11752643 | T | C | 0.02639 | 0.0802 | 0.0088 | 3.96E-19 |
| rs998584 | A | C | 0.5145 | 0.0293 | 0.0037 | 3.42E-15 |
| rs38855 | A | G | 0.5264 | 0.0187 | 0.0033 | 2.11E-08 |
| rs287621 | T | C | 0.2704 | 0.0222 | 0.0037 | 7.67E-09 |
| rs4719841 | G | A | 0.3826 | 0.0232 | 0.0034 | 8.86E-11 |
| rs11974409 | A | G | 0.8061 | 0.0899 | 0.0042 | 1.40E-100 |
| rs72555385 | G | A | 0.06201 | 0.0749 | 0.0124 | 3.76E-09 |
| rs6995541 | G | A | 0.3219 | 0.0265 | 0.0037 | 1.34E-12 |
| rs1062219 | T | C | 0.4921 | 0.0223 | 0.0034 | 1.69E-09 |
| rs2954022 | C | A | 0.5303 | 0.078 | 0.0033 | 2.20E-113 |
| rs4871624 | G | T | 0.2652 | 0.0254 | 0.0037 | 1.07E-11 |
| rs4921914 | C | T | 0.248 | 0.0353 | 0.004 | 4.87E-17 |
| rs7016529 | C | T | 0.01319 | 0.1911 | 0.014 | 3.57E-35 |
| rs12678919 | A | G | 0.8786 | 0.1702 | 0.0056 | 1.80E-199 |
| rs4738684 | A | G | 0.3522 | 0.0205 | 0.0035 | 8.82E-09 |
| rs7005265 | T | A | 0.297 | 0.0336 | 0.0053 | 1.26E-10 |

| **Supplementary Table 4. One-sample MR with a single instrument and additional adjustment for all three lipid instruments** | | | | | | | |
| --- | --- | --- | --- | --- | --- | --- | --- |
| **Lipid parameter** | **Phenotype** | **Single instrument MR effect size** | **Single instrument MR 95% CI** | **Single instrument MR p-value** | **Multiple instrument MR**  **effect size** | **Multiple**  **instrument MR**  **95% CI** | **Multiple**  **instrument MR**  **p-value** |
| **LDL Cholesterol** | **LV EDV (ml)** | 1.85 | 0.59 to 3.14 | 0.004 | 2.12 | 0.08 to 3.46 | 0.001 |
|  | **LV mass (g)** | 0.81 | 0.11 to 1.51 | 0.023 | 0.77 | 0.04 to 1.51 | 0.037 |
| **Triglycerides** | **LV EF (%)** | -0.52 | -0.92 to -0.13 | 0.011 | -0.54 | -0.99 to -0.10 | 0.019 |
|  | **LV mass (g)** | 1.37 | 0.45 to 2.3 | 0.004 | 1.54 | 0.50 to 2.59 | 0.004 |

| **Supplementary Table 5. One-sample MR adjusted for all potential confounders** | | | | |
| --- | --- | --- | --- | --- |
| **Lipid parameter** | **CMR parameter** | **MR effect size** | **MR 95% CI** | **MR p-value** |
| **LDL Cholesterol** | **LV EDV (ml)** | 1.68 | 0.38 to 3.0 | 0.010 |
|  | **LV EF (%)** | 0.06 | -0.25 to 0.37 | 0.710 |
|  | **LV mass (g)** | 0.72 | 0.01 to 1.45 | 0.046 |
| **HDL Cholesterol** | **LV EDV (ml)** | 3.41 | -0.35 to 7.16 | 0.071 |
|  | **LV EF (%)** | 0.44 | -0.47 to 1.35 | 0.338 |
|  | **LV mass (g)** | 0.40 | -1.68 to 2.49 | 0.703 |
| **Triglycerides** | **LV EDV (ml)** | -0.93 | -2.62 to 0.78 | 0.279 |
|  | **LV EF (%)** | -0.56 | -0.98 to -0.15 | 0.008 |
|  | **LV mass (g)** | 1.04 | 0.1 to 1.99 | 0.034 |
| *One-sample MR data following additional adjustment for age at recruitment, sex, BMI, BSA, systolic blood pressure adjusted for anti-hypertensive medication use, physical activity, smoking status, HbA1c and presence of cardiovascular disease; data is presented for change in LV parameter per 1 mmol/L increase in lifetime exposure to lipid parameter* | | | | |

| **Supplementary Table 6: Associations between LV parameters and lipid parameter with phenotypic and genetically-determined lipid level included as covariates** | | | | |
| --- | --- | --- | --- | --- |
| **Phenotype** | **Lipid parameter** | **Effect size** | **95% CI** | **p-value** |
| LV EDV | Measured LDL cholesterol | -3.91 | -4.39 to -3.44 | 1.60E-58 |
| LV EDV | Genetically-determined LDL cholesterol | 5.91 | 4.54 to 7.29 | 3.63E-17 |
| LV EF | Measured LDL cholesterol | 0.24 | 0.12 to 0.35 | 4.18E-05 |
| LV EF | Genetically-determined LDL cholesterol | -0.20 | -0.53 to 0.12 | 2.24E-01 |
| LV mass | Measured LDL cholesterol | -0.52 | -0.79 to -0.26 | 1.11E-04 |
| LV mass | Genetically-determined LDL cholesterol | 1.36 | 0.60 to 2.13 | 5.00E-04 |
| LV EDV | Measured HDL cholesterol | 12.26 | 11.02 to 13.50 | 4.30E-83 |
| LV EDV | Genetically-determined HDL cholesterol | -8.96 | -12.82 to -5.10 | 5.44E-06 |
| LV EF | Measured HDL cholesterol | 0.05 | -0.25 to 0.35 | 7.55E-01 |
| LV EF | Genetically-determined HDL cholesterol | 0.39 | -0.54 to 1.32 | 4.12E-01 |
| LV mass | Measured HDL cholesterol | 2.05 | 1.35 to 2.74 | 8.25E-09 |
| LV mass | Genetically-determined HDL cholesterol | -1.97 | -4.14 to 0.20 | 7.48E-02 |
| LV EDV | Measured triglycerides | -5.18 | -5.59 to -4.77 | 1.78E-134 |
| LV EDV | Genetically-determined triglycerides | 4.47 | 2.86 to 6.08 | 5.61E-08 |
| LV EF | Measured triglycerides | 0.23 | 0.13 to 0.33 | 5.16E-06 |
| LV EF | Genetically-determined triglycerides | -0.73 | -1.12 to -0.34 | 2.37E-04 |
| LV mass | Measured triglycerides | -0.46 | -0.69 to -0.23 | 7.73E-05 |
| LV mass | Genetically-determined triglycerides | 1.79 | 0.88 to 2.70 | 1.19E-04 |
| *Models are adjusted for age, sex, body surface area and the first 5 genetic principal components. The effect sizes represent the change in LV parameter for every 1 mmol/L increment in lipid concentration.* | | | | |

| **Supplementary Table 7: One-sample MR results using restricted list of variants in GRS** | | | | |
| --- | --- | --- | --- | --- |
| **Lipid parameter** | **Phenotype** | **MR effect size** | **MR 95% CI** | **MR p value** |
| LDL Cholesterol | LV EDV (ml) | 2.12 | 0.74 to 3.53 | 0.002 |
| LDL Cholesterol | LV EF (%) | -0.06 | -0.39 to 0.26 | 0.695 |
| LDL Cholesterol | LV mass (g) | 1.01 | 0.24 to 1.78 | 0.009 |
| Triglycerides | LV EDV (ml) | -0.61 | -2.26 to 1.08 | 0.469 |
| Triglycerides | LV EF (%) | -0.40 | -0.8 to 0.001 | 0.052 |
| Triglycerides | LV mass (g) | 1.13 | 0.21 to 2.08 | 0.018 |
| *One-sample MR data utilising a weighted genetic risk score built using a restricted list of variants following removal of variants which might potentially influence LV remodeling. The total number of included variants is 69, 81 and 50 for LDL, HDL and triglycerides genetic risk scores, respectively. Data is adjusted for age, sex, BSA and the first five principal components and data is presented as change in LV parameter per 39 mg/dL (1 mmol/L) for LDL and HDL cholesterol and 89 mg/dL for triglyceride increase in lifetime lipid parameter exposure.* | | | | |

| **Supplementary Table 8: Two-sample single instrument and multivariable Mendelian randomization analysis using summary level data** | | | | | | | |
| --- | --- | --- | --- | --- | --- | --- | --- |
| **Lipid parameter** | **Phenotype** | **Single instrument IVW beta** | **IVW confidence**  **interval** | **IVW**  **p-value** | **Multivariable MR IVW beta** | **Multivariable MR IVW confidence interval** | **Multivariable MR IVW p-value** |
| **LDL Cholesterol** | **LV EDV (ml)** | 1.62 | 0.32 to 2.91 | 0.014 | 1.90 | 1.13 to 2.67 | <0.0001 |
|  | **LV EF (%)** | 0.04 | -0.17 to 0.25 | 0.705 | 0.11 | -0.06 to 0.28 | 0.207 |
|  | **LV mass (g)** | 0.66 | 0.1 to 1.22 | 0.021 | 0.55 | 0.13 to 0.96 | 0.010 |
| **Triglycerides** | **LV EDV (ml)** | -0.43 | -1.73 to 0.86 | 0.512 | -1.1 | -2.21 to 0.01 | 0.052 |
|  | **LV EF (%)** | -0.30 | -0.66 to 0.06 | 0.106 | -0.23 | -0.47 to 0.01 | 0.060 |
|  | **LV mass (g)** | 0.61 | 0.04 to 1.18 | 0.036 | 0.61 | 0.01 to 1.20 | 0.047 |
| *For two-sample MR the change in LV parameter reflects an increase per 34 mg/dL (0.87 mmol/L) 15 mg/dL (0.38 mmol/L) and 90 mg/dL (1.02 mmol/L) increase in LDL cholesterol, HDL cholesterol and triglycerides, respectively.* | | | | | | |  |

| **Supplementary Table 9: Two-sample MR with MR-Egger, weighted median and weighted mode methods** | | | | | | | | | | |
| --- | --- | --- | --- | --- | --- | --- | --- | --- | --- | --- |
| **Lipid parameter** | **CMR parameter** | **Egger effect size** | **Egger confidence interval** | **Egger p-value** | **Weighted median effect size** | **Weighted median confidence interval** | **Weighted median p-value** | **Weighted mode effect size** | **Weighted mode confidence interval** | **Weighted mode p-value** |
| **LDL Cholesterol** | **LV EDV (ml)** | 1.90 | 0.2 to 3.59 | 0.029 | 2.12 | 0.44 to 3.8 | 0.014 | 1.93 | 0.28 to 3.58 | 0.022 |
|  | **LV EF (%)** | 0.12 | -0.18 to 0.42 | 0.422 | 0.14 | -0.19 to 0.48 | 0.407 | 0.40 | -0.52 to 1.32 | 0.395 |
|  | **LV mass (g)** | 0.32 | -0.6 to 1.25 | 0.497 | 0.58 | -0.27 to 1.43 | 0.182 | 0.14 | -0.18 to 0.46 | 0.402 |
| **HDL Cholesterol** | **LV EDV (ml)** | 1.00 | -1.15 to 3.15 | 0.363 | 1.26 | -0.65 to 3.17 | 0.195 | 1.48 | -0.38 to 3.34 | 0.119 |
|  | **LV EF (%)** | 0.23 | -0.26 to 0.73 | 0.359 | 0.26 | -0.15 to 0.67 | 0.209 | 0.32 | -0.12 to 0.76 | 0.158 |
|  | **LV mass (g)** | 0.81 | -0.12 to 1.73 | 0.087 | 0.36 | -0.62 to 1.35 | 0.472 | 0.63 | -0.39 to 1.65 | 0.227 |
| **Triglycerides** | **LV EDV (ml)** | 0.14 | -1.82 to 2.1 | 0.886 | -0.96 | -3.04 to 1.12 | 0.365 | -0.32 | -2.41 to 1.76 | 0.762 |
|  | **LV EF (%)** | -0.27 | -0.86 to 0.33 | 0.380 | 0.05 | -0.44 to 0.53 | 0.851 | 0.11 | -1.1 to 1.33 | 0.858 |
|  | **LV mass (g)** | 0.09 | -0.74 to 0.93 | 0.824 | 0.33 | -0.75 to 1.4 | 0.549 | -0.18 | -0.67 to 0.32 | 0.487 |
| *For two-sample MR the change in LV parameter reflects an increase per 34 mg/dL (0.87 mmol/L) 15 mg/dL (0.38 mmol/L) and 90 mg/dL (1.02 mmol/L) increase in LDL cholesterol, HDL cholesterol and triglycerides, respectively.* | | | | | | | | | | |

| **Supplementary Table 10: MR pleiotropy residual sum and outlier (MR-PRESSO) analysis for horizontal pleiotropy** | | | | | | | | | |
| --- | --- | --- | --- | --- | --- | --- | --- | --- | --- |
| **Lipid parameter** | **Phenotype** | **Global p-value** | **Original beta** | **Original CI** | **Original p-value** | **Corrected beta** | **Corrected CI** | **Corrected p-value** | **Distortion p-value** |
| **LDL Cholesterol** | **LV EDV** | <0.0001 | 1.62 | 0.32 to 2.91 | 0.014 | 1.61 | 0.46 to 2.76 | 0.007 | 0.788 |
|  | **LV EF** | 0.3824 | 0.04 | -0.17 to 0.25 | 0.705 | No outliers |  |  |  |
|  | **LV mass** | 0.0314 | 0.66 | 0.1 to 1.22 | 0.021 | No outliers |  |  |  |
| **HDL cholesterol** | **LV EDV** | <0.0001 | 1.16 | -0.07 to 2.39 | 0.065 | 0.98 | -0.41 to 2.37 | 0.169 | 0.38 |
|  | **LV EF** | 0.0042 | 0.18 | -0.08 to 0.44 | 0.184 | 0.17 | -0.11 to 0.44 | 0.233 | 0.764 |
|  | **LV mass** | 0.0018 | 0.32 | -0.26 to 0.89 | 0.279 | 0.32 | -0.37 to 1.01 | 0.37 | 0.711 |
| **Triglycerides** | **LV EDV** | 0.0356 | -0.43 | -1.73 to 0.86 | 0.512 | -0.48 | -1.85 to 0.89 | 0.496 | 0.39 |
|  | **LV EF** | 0.0417 | -0.3 | -0.66 to 0.06 | 0.106 | No outliers |  |  |  |
|  | **LV mass** | 0.1318 | 0.61 | 0.04 to 1.18 | 0.036 | No outliers |  |  |  |

| **Supplementary Table 11: MR-Steiger analysis** | | | | | |
| --- | --- | --- | --- | --- | --- |
| **Exposure** | **Outcome** | **SNP r^2^ for exposure** | **SNP r^2^ for outcome** | **Directionality test** | **MR-Steiger p-value** |
| **LDL Cholesterol** | **LV EDV (ml)** | 0.0879 | 0.0106 | TRUE | 1.30 x10^-138^ |
|  | **LV EF (%)** | 0.0879 | 0.0061 | TRUE | 7.50 x10^-174^ |
|  | **LV mass (g)** | 0.0879 | 0.0078 | TRUE | 1.03 x10^-158^ |
| **Triglycerides** | **LV EDV (ml)** | 0.0570 | 0.0057 | TRUE | 3.27 x10^-96^ |
|  | **LV EF (%)** | 0.0570 | 0.0057 | TRUE | 7.03 x10^-96^ |
|  | **LV mass (g)** | 0.0570 | 0.0053 | TRUE | 1.48 x10^-99^ |

| **Supplementary Table 12: The variance of phenotypic LV parameters explained by observed lipid measurements and lipid genetic risk scores** | | |
| --- | --- | --- |
| **Lipid parameter** | **Phenotype** | **R^2^** |
| Variance of LV phenotypes explained by measured lipids | | |
| LDL Cholesterol | LV EDV | 0.35% |
|  | LV EF | 0.06% |
|  | LV mass | 0.10% |
| HDL Cholesterol | LV EDV | 6.46% |
|  | LV EF | 1.68% |
|  | LV mass | 14.48% |
| Triglycerides | LV EDV | 0.60% |
|  | LV EF | 0.18% |
|  | LV mass | 5.42% |
| Variance of LV phenotypes explained by GRS | | |
| LDL Cholesterol | LV EDV | 0.01% |
|  | LV EF | 0.00% |
|  | LV mass | 0.00% |
| HDL Cholesterol | LV EDV | 0.01% |
|  | LV EF | 0.01% |
|  | LV mass | 0.00% |
| Triglycerides | LV EDV | 0.00% |
|  | LV EF | 0.04% |
|  | LV mass | 0.01% |

| **Supplementary Table 13. Interaction analysis of the statin therapy on the relationships between the genetic risk score for LDL cholesterol and LV parameters** | | | | |
| --- | --- | --- | --- | --- |
| **Lipid parameter** | **CMR parameter** | **Interaction effect size** | **Interaction standard error** | **Interaction p-value** |
| **LDL Cholesterol** | **LV EDV (ml)** | 0.26 | 0.50 | 0.610 |
|  | **LV EF (%)** | -0.19 | 0.12 | 0.110 |
|  | **LV mass (g)** | -0.07 | 0.27 | 0.788 |

Supplementary Figure 1

Supplementary Figure 2

Supplementary Figure 3

Supplementary Figure 4

Supplementary Figure 5
