# Supplementary material for: The Effect of Blood Lipids on the Left Ventricle: A Mendelian Randomization Study
Source: J Am Coll Cardiol. Author manuscript; Available in PMC 2022 Aug 6. (PMC7613249; doi:10.1016/j.jacc.2020.09.583)
Supplement: Appendix [file EMS151235-supplement-Appendix.pdf]

JACC VOL. 76, NO. 21, 2020
NOVEMBER 24, 2020:2477–88

APPENDIX For supplemental tables and figures, please see the online version of this paper.
